# Mechanism of Consistent Gyrus Formation: an Experimental and Computational Study

**DOI:** 10.1038/srep37272

**Published:** 2016-11-17

**Authors:** Tuo Zhang, Mir Jalil Razavi, Xiao Li, Hanbo Chen, Tianming Liu, Xianqiao Wang

**Affiliations:** 1 Brain Decoding Research Center and School of Automation, Northwestern Polytechnical University, 710072, China; 2 Department of Computer Science and Bioimaging Research Center, The University of Georgia, Athens, GA, 30602, USA; 3 College of Engineering, The University of Georgia, Athens, GA, 30602, USA.

## Abstract

As a significant type of cerebral cortical convolution pattern, the gyrus is widely preserved across species. Although many hypotheses have been proposed to study the underlying mechanisms of gyrus formation, it is currently still far from clear which factors contribute to the regulation of consistent gyrus formation. In this paper, we employ a joint analysis scheme of experimental data and computational modeling to investigate the fundamental mechanism of gyrus formation. Experimental data on mature human brains and fetal brains show that thicker cortices are consistently found in gyral regions and gyral cortices have higher growth rates. We hypothesize that gyral convolution patterns might stem from heterogeneous regional growth in the cortex. Our computational simulations show that gyral convex patterns may occur in locations where the cortical plate grows faster than the cortex of the brain. Global differential growth can only produce a random gyrification pattern, but it cannot guarantee gyrus formation at certain locations. Based on extensive computational modeling and simulations, it is suggested that a special area in the cerebral cortex with a relatively faster growth speed could consistently engender gyri.

Deciphering the characteristic folding pattern of the cerebral cortex is still an important but challenging issue. During the third trimester of gestation, the human cerebral cortex experiences rapid growth and begins to form a wrinkled appearance^1^,^2^. The cerebral cortex of the primate brains eventually becomes highly convoluted and folds itself into a complicated pattern. The convolution pattern of the cerebral cortex looks closely related to the architectural, connectional, and functional specialization of the cortical surface^3^. The role of cortical folding in the function of the brain has not been completely understood yet, although many studies have shown that abnormal folding may lead to brain malformations including Schizophrenia, Autism, Lissencephaly and Polymicrogyria^4^,^5^,^6^,^7^. Clarifying the cortical folding process and its relationship to a healthy brain function could open new windows towards diagnosis and treatment of a disordered brain. Evidence has also shown that the convolution pattern of the cerebral cortex can predict its cytoarchitecture^8^. Therefore, quantitative descriptions of convolution pattern and fundamental understanding of the underlying mechanisms have emerged as two important research goals for a long time^9^,^10^,^11^.

The grooves in the convoluted brain are called sulci, and the bulging ridges between them are called gyri. The gyri-centric representation of cortical folding has attracted many studies related to cortical convolutions^12^, as they are the most basic and commonly preserved pattern across species. To unveil the underlying mechanisms, many hypotheses have been proposed from a variety of perspectives and scales. For example, some studies reported that cortical folding may be caused by external or internal causes, such as cranial constraint^13^,^14^ and axon maturation^15^,^16^,^17^. In some other works, differential growth at the cellular level^18^,^19^,^20^ has been deemed the driving force for gyrification. In the differential growth hypothesis, without any other external or internal constraints or supports, a faster growth rate of the outer layer compared with the inner layer of the brain acts as a driving mechanism for gyrification. These hypotheses were supported not only by experimental observation^18^ but also computational modeling^3^,^21^,^22^,^23^. Rather than being a randomly varied pattern, the primary gyro-sulcal layout of a species is preserved across subjects; although more elaborate convolution (secondary and tertiary convolution) patterns are variable^24^,^25^. The reason for this reproducibility has not been reported in those available works. Recent interesting genetic studies report a number of discoveries towards potential fundamental regulators^26^,^27^,^28^,^29^. For example, radial/lateral cortical expansion is reported^28^ to cause gyri formation, but more support is still needed to bridge the gap between the genetic foundation and phenotypic response. The degree of cortical folding has been reported to uniformly scale across all cortices as a function of the product of cortical surface area and the square root of cortical thickness^30^. It suggests that geometric parameters should be considered to analyze the mechanical specification of a developing brain. With this idea in mind, the past several years have witnessed computational modeling evolving into an influential technique for validating or verifying experimental results, supporting and augmenting analytical models^31^. For example, finite element (FE) analysis has offered noteworthy insights into the growth, instability, morphogenesis, and functions of the brain^32^,^33^. Recent 2D and 3D FE models have been designed and implemented to elucidate the role of mechanical parameters during brain development^23^,^31^. Interestingly, results show that morphological abnormalities related to the developing brain can be presented by mechanical models^34^,^35^. In a recent study, considerable advancement has been made with respect to the modeling of the morphological evolution of the developing brain, but many questions remain unanswered which beg for further analytical, computational, and experimental investigations. For example, why are the primary cortical convolutions within each species highly correlated and consistent rather than random, and what factors count for this consistency as regulators? Therefore, here we would like to attempt to answer, experimentally and computationally, why special areas in the brain form gyri, what factors could affect this mechanism, and how these parameters control the consistency of patterns.

For the experimental analysis, we adopt macro-scale structural T1/T2 weighted MRI data to study the effect of growth rate of the cortical surface on gyrus-sulcus formation. The advantage of the structural MRI data is that gyrus-sulcus convolution patterns can be clearly revealed via a 3D cortical surface reconstruction technique^36^. However, as the cortical growth rate is a micro-scale characteristic and is difficult to measure in a direct way, we thus use the cortical thickness measured on the structural MRI data instead to infer the relationship between the growth rate and the gyro-sulcal pattern. The relationship between gyro-sulcal patterns and cortical thickness has been reported in many previous studies^37^,^38^,^39^. In those studies, gyral regions are found to have thicker cortices as well as more neurons than sulcal regions. However, this relationship was usually investigated on matured brains because of their elaborated folding patterns which are difficult to find on the fetal cortex. Also, this relationship does not directly reveal the causality between the two factors^40^. In recent genetic studies^41^,^42^, genes such as Trnp1 and Cdk4/CyclinD1 are reported to regulate the neuron migration process and cause regionalized cortical folding patterns. For example, low expression of Trnp1 in the ventricular zone results in more neurons migrating to the corresponding cortical plate, and further causes the radial and lateral expansion of the cortex which can engender convex folding patterns. Based on these aforementioned works, we infer that more gene-regulated neuron migration will lead to faster cortical growth, which further results in convex folding patterns (Fig. S1(b–d)). In order to support this hypothesis from the imaging data perspective, we use longitudinal fetal MRI atlas data in this work to measure the growth rate of different folding patterns.

For the computational analysis, we use a 3D plate model consisting of a double-layer soft tissue to represent a small piece of the brain (Fig. S1(a)). The top layer represents the developing cortical plate (cortex) and the bottom layer is the core of the brain which is considered as a simple organization of the subplate, intermediate zone, and ventricular zone^35^. The Dynamic-Explicit solver in the ABAQUS software is implemented to perform morphological evolution of the growing brain. Special areas in the top layer, in which the growth rate and stiffness are different from other cortical regions, are applied to study the structural mechanism for gyri formation.

## Results

### Experimental analysis results

First, we extract data for the thickness of the cortices across a variety of cortical regions from ten subjects in HCP dataset as well as their parcellation annotations (Destrieux parcellation scheme). The parcels which are explicitly identified as gyri or sulci are used. The averaged cortical thickness within a parcel is computed and reported in Table 1. For each parcel, the average is computed over vertices and subjects. On average, vertices in gyral regions across all subjects have cortices (2.91 ± 0.89 *mm*), which are significantly thicker than those in sulcal cortices (2.91 ± 0.81 *mm*) with a small *p*-value (1.01 × 10^−29^) for the unpaired *t*-test. The null hypothesis is that the two groups have equal means with equal variances. This discrepancy is also confirmed by thickness values averaged across individuals (Tables S1, S2, S3, S4, S5, S6, S7, S8, S9 and S10). Referring to the assumption that faster growth rates in the developing stage might result in thicker cortices in the matured brain, these results imply that cortices with faster growth rates have a greater chance to give rise to gyri formation.

**Figure 1:**
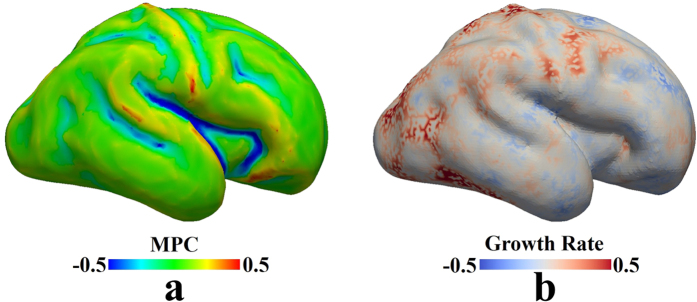
Comparison between cortical folding patterns and cortical growth rates. The white matter surface of the 31^st^ week fetal brain atlas is used as a reference. (**a**) Maximal principal curvature (MPC) measured on the surface is used to represent the gyro-sulcal folding patterns. Convex patterns have positive MPC values whereas concave patterns have negative values. (**b**) Growth rate is computed vertex-wise based on the cortical thickness values from the 25^th^ week fetal brain to the 31^st^ week fetal brain. The spherical registration method^43^ is used to warp the other white matter surfaces (from the 25^th^ week to the 30^th^ week) to the 31^st^ week one and correspondences between vertices across time are established accordingly. Growth rate is defined as the slope of the linear regression fit to the cortical thickness curve.

Further, the change of the cortical thickness over time on the longitudinal fetal MRI atlas data provides more straightforward evidence towards the assumption that faster growth rates consistently engender gyral regions. Specifically, we first obtain the cortical thickness for each vertex on white matter surfaces from the 25^th^ week to the 31^st^ week. Second, we use the spherical registration method^43^ to warp white matter surfaces from the 25^th^ week to the 30^th^ week to the 31^st^ week surface. Based on these results, correspondence among vertices on surfaces over time is established. Then, we use the linear regression model to fit the cortical thickness curve for each vertex on the 31^st^ week surface. The slope of the regression fit is used to represent the growth rate of the cortex. Cortices with fast growth rates are expected to have large slope values. It is noted that we choose fetal atlases from the 25^th^ week to the 31^st^ week and map the results to the 31^st^ week because the 25^th^ week cortex is smooth whereas many major gyri and sulci are identified on the 31^st^ week surface^44^ (see Fig. 1(b)), such as the central sulcus, precentral gyrus, postcentral gyrus, and the gyri and sulci on the temporal lobe. On the 31^st^ week surface, we compute the maximal principal curvature (MPC, Fig. 1(a)) and use it as a descriptor of gyro-sulcal patterns. Generally, gyri have positive MPC values whereas sulci have negative values. Therefore, we separate vertices into two groups, those with positive MPC values and those with negative values. We conduct *t*-tests on the two groups to test the alternative hypothesis that the positive group mean is not equal to the negative group mean. We also conduct unpaired right tail *t*-tests to test the alternative hypothesis that the positive group mean is greater than the negative group mean. The *p*-values are reported in Table 2. It is worth noting that 51% of the vertices have their *R*^2^ values, used to evaluate the performance of regression, higher than 0.6, indicating the linear model is qualified to a certain degree. It also suggests that not all cortical thickness curves are well predicted by linear regression model. Other models such as exponent model could potentially better predict the cortical growth. Lower *R*^2^ values could also be attributed to other reasons such as registration errors, though the selection of model and improvement of registration method are not in the scope of this work. Therefore, with the choice of linear model, we only perform the abovementioned statistical analysis in Table 2 on ‘trustworthy’ vertices, whose *R*^2^ values are above 0.6. It is seen in Table 2 that gyral patterns (positive MPC vertex group) have greater cortical growth rates than sulcal patterns (negative MPC vertex group). This result provides more direct evidence to the assumption that faster growth rates engender gyral regions.

In summary, those experimental results suggest that thicker cortical regions consistently engender gyral patterns. They further imply that heterogeneous growth in cortical regions (rather than a global homogeneous differential growth) with faster growth rates might consistently produce a gyral pattern at that location. Therefore, in the following computational experiments, heterogeneous growth rates are assigned to different regions (see the special area in Fig. S2) to further explore how the consistent gyrus forms at a fast growing location.

### Computational Results

#### Homogeneous growth in the cortex

**Figure 2:**
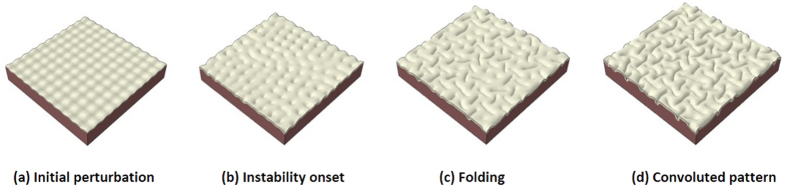
Morphological evolution of a growing brain model. (**a**) Initial perturbation before simulation; (**b**) Instability initiation; (**c**) Folding after instability; (**d**) Convoluted pattern.

In order to single out the determining factors in gyri formation, we first perform a series of simulations with homogeneous growth in the cortex and consider them as control studies. Figure 2 shows a typical evolution process of a growing brain model with a homogeneously growing cortex: (a) first the model starts to expand in both the normal and vertical directions; (b) after a certain amount of growth, instability is engendered due to the substantial compressive stresses in the confined but growing cortex; (c) the cortex after instability starts to wrinkle and then fold, forming gyri and sulci; (d) with the continuation of growth, folds become more convoluted and self-contact occurs between gyri. Homogeneous growth in the cortex produces convolution patterns with gyri and sulci as we expect, but the gyri or sulci formation sites in the convoluted model seem unpredictable and this formation process may be considered as a random process.

**Figure 3:**
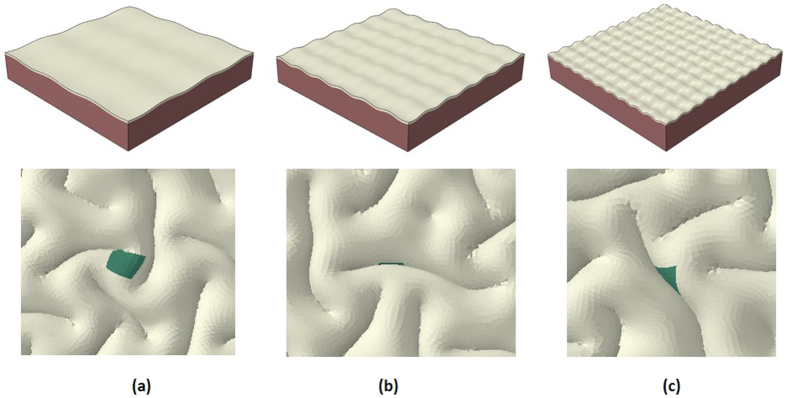
Different initial perturbations cause the center of the cortex to be located in different positions. (**a**) On the top of a gyrus; (**b**) On a sulcus; (**c**) On the wall of a gyrus.

To confirm the randomness of the gyri or sulci sites in the model with a homogeneous cortex, we perform a series of simulations with the same model specifications (growth rate, geometric parameters and material properties) except for small initial perturbations. Figure 3 shows that different initial perturbations lead to the formation of gyri and sulci in different locations, in contrast to Fig. 2. It can be seen that the qualitative or overview features are almost the same in the three models but the locations for gyri formation are quite different and random by tracing the center area of the cortex. The center area of the cortex can be on a gyrus, the wall of a gyrus, or even a sulcus as shown in the subplots of Fig. 3. These findings suggest that homogeneous growth in the cortex fails to cause consistent gyrus or sulcus formation on a special site. The size of folds is not a target of this study, but for finding a relationship between the size of folds and cortex thickness in both imaging data and FE model please check references^23^,^45^.

#### Heterogeneous growth in the cortex

**Figure 4:**
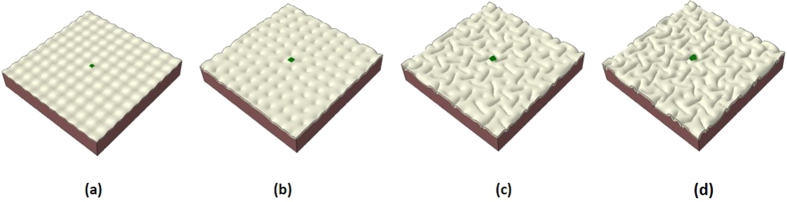
Morphological evolution of a growing brain model with heterogeneous growth in the cortex. (**a**) Initial perturbation before simulation; (**b**) Instability initiation; (**c**) Folding after instability; (**d**) Convoluted pattern. The special area is on the top of a gyrus after convolution.

Following the control study, we test our hypothesis that heterogeneous growth in the cortex can regulate the site of gyri or sulci formation by considering that a small area in the center of cortex section grows faster than other parts of the cortex. In computational models, we suppose that the special area grows 1.2 times faster than other cortex areas. This value is estimated from the experimental data which indicates that the thickness of gyri is roughly 1.2 times the thickness of sulci as shown in Table 1. Figure 4 shows a typical evolution process of a growing brain model with a special area in the cortex growing faster than the rest of the cortex. It experiences a similar evolution process as the one discussed in Fig. 2, but there is a gyrus formed in the special area.

An interesting question arises that what the role of the special area in the cortex plays on gyri or sulci formation. In other words, how does the introduction of the special area with a faster growth rate change the phase diagram of gyri formation on the special area when other parameters are kept intact, e.g. initial perturbations, growth ratio of the cortex, even mesh size of the model? Based on the comparison between the homogeneous growth and heterogeneous growth results (Figs 2 and 4), it is inferred that a gyrus will be formed in areas which have more growth in contrast to other areas of the cortex.

In order to validate the aforementioned hypothesis, we run a substantial amount of cases (fifty cases with homogeneous growth and fifty cases with heterogeneous growth). Because the special region in the heterogeneous experiment is located in the center of the cortex, the folding patterns of the center area in these experiments are collected and shown in Table 3. It can be seen that there is a very high possibility (94% chance) to form a gyrus in the special area under heterogeneous growth while the formation of gyri, sulci or side of gyri (banks) on the center area with the homogenous growth models share almost even chances.

#### Effect of stiffness

**Figure 5:**
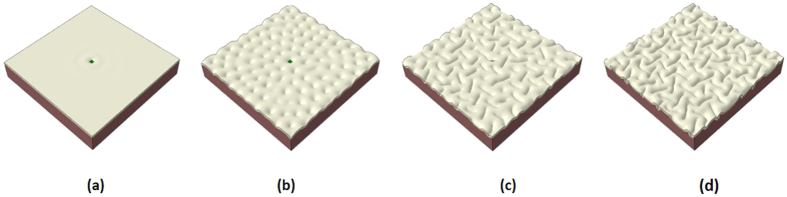
Morphological evolution of a growing cortex with the stiff area in the center. (**a**) Stiff area starts to move downward; (**b**) Instability initiation; (**c**) Folding after instability; (**d**) Convoluted pattern.

Another parameter worthy of studying is the stiffness of the special area, which is hypothesized to play an important role on gyri formation^34^. In the model, we let the cortex have a fixed, homogeneous growth rate but the shear modulus of the special area in the cortex is higher than other parts of the cortex. In order to mimic real brain growth, we define the shear modulus of the special area as a growth-dependent factor, which means the shear modulus of the special area at the beginning of the growth is the same as the main cortex and it will increase step by step until the final value is two times higher than the initial value in the cortex. As what we did in the previous case study, we perform plenty of computational cases only with different initial perturbations in the models. Results interestingly show that stiffness increase in the special area always leads to sulci formation on the special area, suggesting a possible mechanism for sulci formation. Figure 5 shows the morphological evolution of the growing cortex with a stiff special area in the center. When the stiffness of the special area is increased to 4 or 8 times that of the other regions, the same result of sulci formation in the special area site is observed. It is known that the creation of cortical layers follows an inside-out order^46^. The earlier deeper layers (layer V and VI) contain pyramidal neurons which give rise to corticocortical axons and axons connecting the cortex and subcortical structures^47^,^48^, while the older layers (layers II, III and IV) contain numerous stellate neurons and smaller pyramidal neurons, which give rise to intracortical axons^49^. Because it has been reported^38^,^39^ that gyral regions have thicker, deeper layers while sulcal regions have relatively thinner, superficial ones, it is intuitive to associate these reports with our results that stiffer cortices consistently produce sulci. Therefore, we postulate that during the formation of superficial layers, the greater stiffness of the special areas mimicking more intracortical axons and stellate neurons in special areas may induce further sulcal region formation within a certain context, which adds to our previous understanding that denser fiber connections (mainly comprising corticocortical and cortico-subcortical axons) could induce gyri-sulci formation.

## Conclusion

In this work, we employ experimental data analysis and computational modeling to study the mechanism of consistent gyri formation. From the experimental data analysis results, we confirm that gyri have significantly thicker cortices than sulci. The gyral thickness growth rate is also found to be faster than the sulcal growth rate upon analysis of longitudinal fetal brain data over a period of time (25–31 pcw). Those observations are subsequently reproduced in computational studies. Computational results show that the widely known differential growth theory can only produce unregulated convolution and it cannot predict a gyrus at a certain location, whereas consistent and reproducible convolution patterns on the cerebral cortex are regulated by regional growth heterogeneity, and special areas with a faster growth rate consistently engender gyri. Previous studies show that elliptical brains fold mainly in the longitudinal direction, while rounder brains prefer to fold in the transverse direction. Results reveal that gyrification is greatly controlled by the variability of brain profile. Curvature postpones formation of instability while in a heterogeneous system instabilities occur first in regions of lowest curvature. However, in previous studies there is no exact answer to the question: what is the dominating factor for gyrus formation if the curvature is constant in a special region? We can observe that different regions with the same initial curvature before folding could develop both gyri and sulci after folding. Therefore, although initial curvature or perturbation affects the pattern selection, these factors alone cannot guarantee exact formation site of gyri or sulci. In our simulation results we can also see that initial perturbation in homogeneous growth cases influence the patterns but cannot determine the exact sites of gyri or sulci since the perturbation value is negligible in comparison with the size of the model. However, in heterogeneous growth, different initial perturbations lead to different folding patterns but we can consistently observe the formation of gyri in these special sites with a relatively large growth rate. The heterogeneous growth might be controlled by regional differentiation of RGCs in the early stage of development in the fetal brain. Computational results also show that increased stiffness of a special area on the brain model could induce sulcal shape formation within a specific context. These findings may provide new clues to brain diseases and malformations such as Schizophrenia, Autism, Lissencephaly, and Polymicrogyria in the future.

## Methods

### Human Fetal Brain Atlas Dataset

This study uses publicly available datasets (http://www.brain-development.org/). Generally, this dataset includes T2 templates and tissue probability maps (for the brain mask, cortex, hemispheres, cerebrospinal fluid [CSF], and ventricles) for ages between 23–37 weeks of gestation. To create the 4D probabilistic atlas, 142 T2-weighted fast-spin echo images are acquired on 3 T Philips Intera system with MR sequence parameters TR = 1712 *ms*, TE = 160 *ms*, flip angle 90° and voxel sizes 0.86 × 0.86 × 1 *mm*. The atlas construction is described in detail elsewhere^50^.

### Human Connectome Project Dataset

The Human Connectome Project (HCP) dataset includes diffusion MRI data of 64 subjects obtained from the quarter 1 (Q1) session (http://www.humanconnectome.org/). Structural MRI (sMRI) data in this dataset is used to compute the distribution of cortical thickness across regions and subjects. Imaging parameters are TR: 2400 ms, TE: 2.4 ms, Flip Angle: 8 deg, FOV: 224 mm × 224 mm, voxel size 0.7 mm isotropic; T2 weighted MRI: TR: 3200 ms, TE: 565 ms, Flip Angle: variable, FOV: 224 mm × 224 mm, voxel size 0.7 mm. The preprocessing steps of the sMRI data, including skull removal, tissue segmentation, surface reconstruction, structural attribute (including cortical thickness) computation and labelling etc., are conducted via the HCP standard pipeline via FSL and Freesurfer^51^, and those pre-processed results are all included in the released package.

### Imaging Data Analysis Methods

Generally, HCP dataset provides high resolution sMRI data of a large group of matured humans. It is used to study the relationship between cortical thickness and convolution on the cortices for entire populations. Essentially, the growth rate is, on the other hand, a dynamic measurement, and thus we adopt the longitudinal fetal brain atlas data to study the cortical thickness variation over time.

The surface-based parcellation (Destrieux parcellation scheme) is obtained on HCP subjects via the method in the FreeSurfer toolkit^52^. Cortical thickness attributes of the vertices on the surface are retrieved from the HCP dataset. The cortical thicknesses are measured as the distances between gray matter surfaces and white matter surfaces. As the Destrieux parcellation scheme segments a cortex into gyri and sulci, we separately measure the averaged cortical thickness values on gyri parcels and sulci parcels, and make comparisons between them^52^.

The human fetal brain atlas dataset is composed of longitudinal structural MRI data, and thus the change of cortical thickness across time provides more direct evidence to study the difference between gyral and sulcal regions in terms of cortical growth.

Generally, we calculate the cortical thickness on the surface for each time point. Then, we establish the correspondence of surface vertices over time and use the linear model to fit the cortical thickness curve for each vertex.

**Figure 6:**
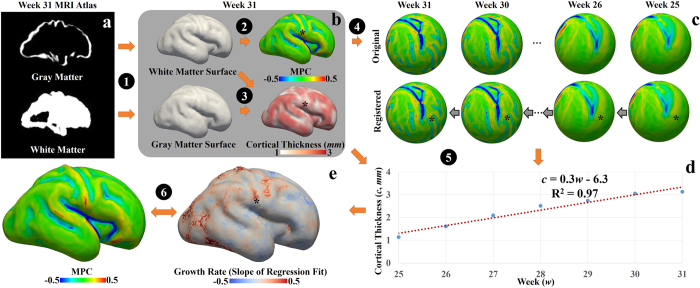
(**a**) MRI atlas of the 31^st^ week fetal brain. (**b**) Structural MRI data processing of one-time point (the 31^st^ week brain is used as an example). Step 1: White matter surface and gray matter surface are reconstructed from the atlas via FreeSurfer. Step 2: maximal principal curvatures (MPCs) are obtained on the white matter surface. Step 3: Cortical thickness is obtained based on the white matter surface and gray matter surface. These processing steps are applied to surfaces of other time points. (**c**) Establishment of correspondence of vertices across time course. Step 4: The spherical registration method^43^ is used to warp white matter surfaces of all time points to the same spherical space (the 31^st^ week). Black stars in (**b**) and (**c**) illustrate a vertex on the 31^st^ week white matter surface and the corresponding vertices on the other surfaces. (**d**) Cortical thickness values of the vertex highlighted by the black star at different time points. The linear regression model is used to fit the thickness curve. The slope of the regression fit is used as a measurement of the growth rate of the cortex over time. (**e**) Growth rate is mapped to the 31^st^ week white matter surface. Step 6: Joint analysis of MPC map and growth rate map.

Specifically, we first conduct a within-time-point process (Fig. 6(a,b)). Segmentation is performed on the atlas using probabilistic white matter, cortex, and CSF maps as initializations. White matter surfaces and gray matter surfaces are reconstructed (Step 1) via the FreeSurfer toolkit^53^,^54^. Maximal principal curvature (MPC) is calculated on the white matter surface (Step 2). MPC is used to represent cortical folding patterns. Generally, a convex cortex section has a positive MPC value, whereas a concave cortex section has a negative MPC value. Cortical thickness for a vertex on the white matter surface is defined as the distance between this vertex and the closest vertex on the gray matter surface (Step 3). Second, we perform a cross-time study. In order to conduct a longitudinal study of the cortical thickness, we need the correspondence for vertices over time. Spherical registration^43^ is adopted to warp surfaces of all time points to the 31^st^ week spherical space (Fig. 6(c)). Because of the huge variation between the 25^th^ week surface and the 31^st^ week surface, we register the surface to the one next to it on the time course (e.g., the 25^th^ week surface to the 26^th^ week surface and so on). The corresponding vertices on the other surfaces to the one on the 31^st^ week surface (black dots) are determined by successively tracing the deformation fields backward. Next, we use a linear regression model to fit the cortical thickness curve for each vertex (Step 5 & Fig. 6(d)). The slope of the regression fit is used as the profile of the growth rate. A large slope value indicates that the cortex of the given vertex grows with a comparatively fast rate. Finally, the slope of the regression fit is mapped to the 31^st^ week surface. It is jointly studied with the MPC map, which represents the convex-concave folding patterns, to investigate the difference between gyri (convex patterns) and sulci (concave patterns) in terms of the growth rate of the cortical thickness.

### Deformation field and governing equation

To find the deformation field of a growing cortex in a planar bilayer model, a decomposition of deformation gradient theory is carried out. The deformation gradient, ***F***, is represented by a multiplication of the elastic tensor, ***A***, which induces stress and a growth tensor, ***G*** which causes volume change, as depicted in Eq. (1). Although both ***G*** and ***A*** tensors may be incompatible deformations, their multiplication, ***F***, should be a compatible deformation^55^. This basic concept in general is valid for both isotropic and anisotropic growth.


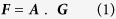


where ***F*** = ∂***x***/∂***X*** and it maps the initial configuration to the grown and current configuration. We consider that the upper layer as the cortex grows isotropically and introduce its growth tensor “***G***” as the identity tensor scaled by the growth multiplier.


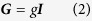


where ***I*** is the identity tensor and *g* is the growth multiplier. Hence, growth rate in all directions (in-plane and out of plane) occurs at an equal rate. Generally, the growth tensor depends on the stress state and deformation field as well as other factors. For simplicity, we assume that the growth tensor is a function with a known spatial distribution, insinuating that all of the information is independent of stresses^56^.

Many biological soft tissues can be modeled by a hyperelastic material with a strain energy function *W(**A***). Also, the elastic deformation of living soft tissues yields little volume change; therefore, the nonlinear response of these materials can be further described by an isotropic incompressible hyperelastic material. The corresponding Cauchy stress ***σ*** can be expressed by an available method^57^


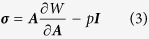


where *p* is a Lagrange multiplier to guarantee incompressibility and ***I*** is the second-order unit vector. In the absence of any body force, mechanical equilibrium imposes


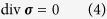


where “div” stands for the divergence operator in the current configuration. There are several proposed material behaviors for hyperelastic materials. Here a simple and common model with a nonlinear neo-Hookean behavior is implemented^32^,^34^,^57^,^58^,^59^,^60^.


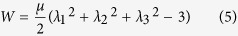


where *μ* is the shear modulus and *λ*_1_, *λ*_2_ and *λ*_3_ are the principal stretches. Deformation and stress fields of a growing model are related to the energy function. This equation follows the fundamental principles of continuum mechanics and is considered as the constitutive relationship of the incompressible hyperelastic material we discuss in this paper.

### Finite Element Model

A three-dimensional (3D) plate model consisting of double-layer soft tissue as a small piece of the brain (Fig. S1(a)) is constructed in this study to investigate the fundamental mechanism of consistent gyri formation during cortical folding. Selection of the flat structure is because the main purpose of this study is to find the effect of the heterogeneous growth and stiffness of cerebral cortex on the gyri formation rather than the effect of the geometry of the model. Two dimensional flat models for cortical folding have been successfully used in recent studies to determine a set of important and effective parameters in the brain folding process, e.g. growth rate, cortex thickness, material property^34^,^61^. Non-flat models have also shown that in addition to these factors, geometrical curvature of the model also plays an important role in gyri formation and pattern selection^21^,^32^. In our model, the top layer represents the developing cortical plate (cortex) and the bottom layer is the core of the brain which is considered as a simple organization of the subplate, intermediate zone, and ventricular zone^59^. The cerebral cortex is a thin (2–4 *mm*)^31^ layer in contrast to the inner core which has a thickness around 50 *mm*^45^. The dimension of the plate is selected to be large enough in comparison with the wavelength of the folded patterns in experiments. The dimension of the model is 300 × 300 × 50 *mm* and the thickness of the cortex is 2 *mm* before growth. Growth in the model is simulated via thermal expansion^23^,^35^,^62^. For understanding the detail of analogy between volumetric growth model and thermal stress model please check reference^62^. Derivations indicate that, at least for isotropic volumetric growth problems, an analysis based on the growth model can be replaced by a thermal stress analysis. For isotropic thermal expansion problems, the thermal deformation gradient can be given by


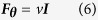


where F_θ_ is the thermal deformation gradient and v is the thermal stretch. Analogy between *g* and *v* from Eqs. 2 and 6 helps to model growth process by thermal expansion^62^. By adjusting the thermal expansion coefficient to the cortex section and increasing temperature in dynamic steps, the cortex expands and then starts to fold.

As discussed in the introduction, the differential growth hypothesis assumes that the outer layer of the brain grows at a faster rate than the inner layer does, which is considered as the driving mechanism of cortical folding^21^.

Figure S1(b,c) shows the biological foundation of the growing brain model with an inferential summary of regulation mechanisms of cortical folding patterns in Fig. S1(d). Generally, radial glial cells (RGCs) with lower levels of Trnp1 could generate basal progenitors (BPs), also known as intermediate progenitor cells (IPCs), and basal radial glial cells (bRGCs). BPs produce neurons while bRGCs provide additional guiding structures inducing faster neuron migration and finally resulting in considerable radial and lateral expansion, i.e. the convex folding pattern suggested in the literature^28^. Therefore, at the cellular level, the distribution difference of RGCs regulates radial expansion of the cortical plate by controlling the amount of migrating neurons. This intriguing regional difference has been confirmed in the cerebral cortex of human fetuses^42^. This biological foundation of the growing brain model lends compelling evidence to support the differential growth hypothesis used in this paper. The Dynamic-Explicit solver in ABAQUS software, which is suitable for large deformation, nonlinear, and quasi-static problems, is implemented to perform the morphological evolution of the growing brain. A series of initial small perturbations are introduced into the models to check the effect of initial perturbations on gyri formation. Deformation patterns after instability are not guaranteed to be exactly symmetric although the initial model is symmetric^63^. Robustness studies have been performed and concluded that as long as the mesh size is small enough, the qualitative features of the models do not depend on the mesh size. To balance the computational cost and accuracy, only the cortex section has a mesh with fine elements. Our previous study has shown that the folded patterns of the model after growth and instability do not depend on the absolute value of the elastic moduli of the cortex and core, rather, they just depend on the core/cortex elastic modulus ratio^35^. Material properties of the cortex and core are kept the same, as it has been shown that there is no significant difference between gray and white matter material properties^64^. We consider the shear modulus of both the cortex and core (μ in Eq. 5) to be 330 *P*^65^. Two types of computational models are considered here: one with a uniform cortex and the other with the cortex having a special area, as shown in Fig. S2. The special area is assumed as a designed factor to induce heterogeneous growth or stiffness in the cortex, thereby unveiling the fundamental mechanism for gyri formation.

## Additional Information

**How to cite this article**: Zhang, T. *et al.* Mechanism of Consistent Gyrus Formation: an Experimental and Computational Study. *Sci. Rep.* **6**, 37272; doi: 10.1038/srep37272 (2016).

**Publisher’s note:** Springer Nature remains neutral with regard to jurisdictional claims in published maps and institutional affiliations.

## Supplementary Material

Supplementary Information

## Figures and Tables

**Table 1:** The averaged cortical thickness (*mm*) of gyri and sulci.

Gyri	Thickness	Sulci	Thickness
G_cingul-Post-dorsal	3.14	S_calcarine	2.19
G_cingul-Post-ventral	2.36	S_central	2.09
G_cuneus	2.15	S_cingul-Marginalis	2.48
G_front_inf-Opercular	3.09	S_circular_insula_ant	3.17
G_front_inf-Orbital	3.08	S_circular_insula_inf	2.80
G_front_inf-Triangul	2.92	S_circular_insula_sup	2.91
G_front_middle	2.90	S_collat_transv_ant	2.88
G_front_sup	3.08	S_collat_transv_post	2.33
G_Ins_lg_and_S_cent_ins	3.31	S_front_inf	2.61
G_insular_short	3.73	S_front_middle	2.57
G_occipital_middle	2.86	S_front_sup	2.73
G_occipital_sup	2.45	S_interm_prim-Jensen	2.61
G_oc-temp_lat-fusifor	3.14	S_intrapariet_and_P_trans	2.46
G_oc-temp_med-Lingual	2.23	S_oc_middle_and_Lunatus	2.34
G_oc-temp_med-Parahip	3.18	S_oc_sup_and_transversal	2.41
G_orbital	2.97	S_occipital_ant	2.63
G_pariet_inf-Angular	2.97	S_oc-temp_lat	2.79
G_pariet_inf-Supramar	2.96	S_oc-temp_med_and_Lingual	2.60
G_parietal_sup	2.56	S_orbital_lateral	2.50
G_postcentral	2.42	S_orbital_med-olfact	2.49
G_precentral	2.97	S_orbital-H_Shaped	2.85
G_precuneus	2.85	S_parieto_occipital	2.45
G_rectus	2.74	S_pericallosal	1.83
G_subcallosal	2.78	S_postcentral	2.39
G_temp_sup-G_T_transv	2.84	S_precentral-inf-part	2.69
G_temp_sup-Lateral	3.27	S_precentral-sup-part	2.62
G_temp_sup-Plan_polar	3.40	S_suborbital	2.83
G_temp_sup-Plan_tempo	2.83	S_subparietal	2.68
G_temporal_inf	3.20	S_temporal_inf	2.82
G_temporal_middle	3.27	S_temporal_sup	2.78
		S_temporal_transverse	2.75
Avg.	2.91 ± 0.89		2.59 ± 0.81

**Table 2:** Slopes of regression fit and unpaired *t*-test between the two groups of vertices, the group of gyral vertices with positive MPC values *vs*. the group of sulcal vertices with negative MPC values, in terms of their cortical growth rates.

Gyral Vertices *Mean* ± *Std*	Sulcal Vertices *Mean* ± *Std*	*p*-value (Right Tail)	*p*-value (Two Tails)
0.35 ± 0.26	0.10 ± 0.26	4.31 × 10^−98^	2.16 × 10^−98^

It is noted that this analysis is only performed on the vertices, whose cortical thickness curves are well fitted by the linear regression model (*R*^2^ ≥ 0.6).

**Table 3:** Statistical results of the gyri and sulci formation in two growth models.

	Homogeneous growth in cortex	Heterogeneous growth in cortex
gyri	14	47
sulci	19	—
banks	17	3
sum	50	50
