# Corrigendum: Mechanism of Consistent Gyrus Formation: an Experimental and Computational Study

**DOI:** 10.1038/srep42269

**Published:** 2017-03-07

**Authors:** Tuo Zhang, Mir Jalil Razavi, Xiao Li, Hanbo Chen, Tianming Liu, Xianqiao Wang

Scientific Reports 6: Article number: 37272; 10.1038/srep37272 published online: 11 17 2016; updated: 03 07 2017.

In the methods section, under the subheading ‘Human Fetal Brain Atlas Dataset’

“Generally, this dataset includes T2 templates and tissue probability maps (for the brain mask, cortex, hemispheres, cerebrospinal fluid [CSF], and ventricles) for ages between 23–37 weeks of gestation. To create the 4D probabilistic atlas, 142 T2-weighted fast-spin echo images are acquired on 3 T Philips Intera system with MR sequence parameters TR = 1712 ms, TE = 160 ms, flip angle 90° and voxel sizes 0.86 × 0.86 × 1 mm. The atlas construction is described in detail elsewhere^50^”.

should read:

“T2 weighted MR images from 80 fetuses with normal brain appearances were used to create the 4D atlas. The age range at the time of scan was 21.7 to 38.7 weeks gestational age (GA), with mean and standard deviation of 29.6 ± 4.6 weeks. The images were acquired on 1.5 T Philips Achieva system with the following parameters: T2 weighted single shot Fast Spin Echo (ssFSE) TR = 15000 ms, TE = 160 ms, flip angle = 90° and voxel size = 1.25 × 1.25 × 2.5 mm. For each subject multiple stacks of images (typically a total of 8) were acquired in approximately transverse, sagittal and coronal planes and the data reconstructed into a single 3D brain volume using the slice-to-volume reconstruction method. The reconstruction voxel size was 1.18 × 1.18 × 1.18 mm. More details can be found in studies from Serag *et al*.^50^”.

In addition, reference 50 was listed incorrectly. The correct reference appears below:

Serag, A. *et al*. Construction of a consistent high-definition spatio-temporal atlas of the developing brain using adaptive kernel regression. *NeuroImage* **59**, 2255–2265 (2012).

