# Supplementary material for: Mechanism of Consistent Gyrus Formation: an Experimental and Computational Study
Source: Sci Rep. 2016 Nov 17;6:37272. doi: 10.1038/srep37272 (PMC5112531; doi:10.1038/srep37272)
Supplement: Supplementary Information [file srep37272-s1.pdf]

Mechanism of Consistent Gyrus Formation: an Experimental and Computational Study

Tuo Zhang[1,2*], Mir Jalil Razavi[3*], Xiao Li[1], Hanbo Chen[2], Tianming Liu[2**], Xianqiao Wang[3**]

[1]Brain Decoding Research Center and School of Automation, Northwestern Polytechnical University, China, 710072; [2]Department of Computer Science and Bioimaging Research Center, The University of Georgia, Athens, GA, 30602; [3]College of Engineering, The University of Georgia, Athens, GA, 30602.
*These authors contributed equally to this work.
**Joint correspondence authors (Email: tliu@uga.edu and xqwang@uga.edu).

Supplemental Materials

Table S1 The averaged cortical thickness (mm) of gyri and sulci on subject #1.

Gyri	Thickness	Sulci	Thickness
G_cingul-Post-dorsal	3.33	S_calcarine	2.22
G_cingul-Post-ventral	2.29	S_central	2.11
G_cuneus	2.10	S_cingul-Marginalis	2.57
G_front_inf-Opercular	3.23	S_circular_insula_ant	3.43
G_front_inf-Orbital	3.21	S_circular_insula_inf	3.20
G_front_inf-Triangul	2.98	S_circular_insula_sup	3.11
G_front_middle	2.93	S_collat_transv_ant	3.09
G_front_sup	3.18	S_collat_transv_post	2.40
G_Ins_lg_and_S_cent_ins	3.36	S_front_inf	2.69
G_insular_short	3.67	S_front_middle	2.66
G_occipital_middle	2.83	S_front_sup	2.71
G_occipital_sup	2.30	S_interm_prim-Jensen	2.55
G_oc-temp_lat-fusifor	3.22	S_intrapariet_and_P_trans	2.37
G_oc-temp_med-Lingual	2.18	S_oc_middle_and_Lunatus	2.24
G_oc-temp_med-Parahip	3.20	S_oc_sup_and_transversal	2.35
G_orbital	3.22	S_occipital_ant	2.54
G_pariet_inf-Angular	2.91	S_oc-temp_lat	2.87
G_pariet_inf-Supramar	2.95	S_oc-temp_med_and_Lingual	2.61
G_parietal_sup	2.39	S_orbital_lateral	2.72
G_postcentral	2.30	S_orbital_med-olfact	2.70
G_precentral	2.93	S_orbital-H_Shaped	3.09
G_precuneus	2.83	S_parieto_occipital	2.49
G_rectus	2.99	S_pericallosal	2.08
G_subcallosal	2.73	S_postcentral	2.38
G_temp_sup-G_T_transv	2.86	S_precentral-inf-part	2.78
G_temp_sup-Lateral	3.42	S_precentral-sup-part	2.64

G_temp_sup-Plan_polar	3.64	S_suborbital	3.16
G_temp_sup-Plan_tempo	2.89	S_subparietal	2.68
G_temporal_inf	3.40	S_temporal_inf	2.79
G_temporal_middle	3.41	S_temporal_sup	2.85
		S_temporal_transverse	2.59
Avg.	2.95±0.85		2.67±0.76

Table S2 The averaged cortical thickness (mm) of gyri and sulci on subject #2.

**Gyri**	**Thickness**	**Sulci**	**Thickness**
G_cingul-Post-dorsal	2.96	S_calcarine	2.12
G_cingul-Post-ventral	2.29	S_central	2.01
G_cuneus	2.13	S_cingul-Marginalis	2.48
G_front_inf-Opercular	3.15	S_circular_insula_ant	3.06
G_front_inf-Orbital	2.87	S_circular_insula_inf	2.88
G_front_inf-Triangul	2.96	S_circular_insula_sup	2.87
G_front_middle	2.94	S_collat_transv_ant	2.85
G_front_sup	3.08	S_collat_transv_post	2.46
G_Ins_lg_and_S_cent_ins	3.13	S_front_inf	2.63
G_insular_short	3.61	S_front_middle	2.73
G_occipital_middle	2.94	S_front_sup	2.77
G_occipital_sup	2.50	S_interm_prim-Jensen	2.77
G_oc-temp_lat-fusifor	3.15	S_intrapariet_and_P_trans	2.60
G_oc-temp_med-Lingual	2.00	S_oc_middle_and_Lunatus	2.45
G_oc-temp_med-Parahip	2.96	S_oc_sup_and_transversal	2.51
G_orbital	2.93	S_occipital_ant	2.61
G_pariet_inf-Angular	3.05	S_oc-temp_lat	2.81
G_pariet_inf-Supramar	2.92	S_oc-temp_med_and_Lingual	2.52
G_parietal_sup	2.66	S_orbital_lateral	2.66
G_postcentral	2.48	S_orbital_med-olfact	2.48
G_precentral	3.03	S_orbital-H_Shaped	2.98
G_precuneus	2.95	S_parieto_occipital	2.58
G_rectus	2.66	S_pericallosal	1.66
G_subcallosal	2.80	S_postcentral	2.33
G_temp_sup-G_T_transv	2.95	S_precentral-inf-part	2.61
G_temp_sup-Lateral	3.27	S_precentral-sup-part	2.68
G_temp_sup-Plan_polar	3.32	S_suborbital	3.03
G_temp_sup-Plan_tempo	2.91	S_subparietal	2.74
G_temporal_inf	3.29	S_temporal_inf	2.90
G_temporal_middle	3.21	S_temporal_sup	2.86
		S_temporal_transverse	2.80
Avg.	2.89±0.89		2.63±0.97

Table S3 The averaged cortical thickness (mm) of gyri and sulci on subject #3.

Gyri	Thickness	Sulci	Thickness
G_cingul-Post-dorsal	3.34	S_calcarine	2.29
G_cingul-Post-ventral	2.66	S_central	2.27
G_cuneus	2.14	S_cingul-Marginalis	2.62
G_front_inf-Opercular	3.18	S_circular_insula_ant	3.31
G_front_inf-Orbital	3.05	S_circular_insula_inf	2.83
G_front_inf-Triangul	2.94	S_circular_insula_sup	3.00
G_front_middle	3.06	S_collat_transv_ant	2.97
G_front_sup	3.16	S_collat_transv_post	2.49
G_Ins_lg_and_S_cent_ins	3.44	S_front_inf	2.71
G_insular_short	3.99	S_front_middle	2.60
G_occipital_middle	3.12	S_front_sup	2.89
G_occipital_sup	2.60	S_interm_prim-Jensen	2.76
G_oc-temp_lat-fusifor	3.31	S_intrapariet_and_P_trans	2.71
G_oc-temp_med-Lingual	2.26	S_oc_middle_and_Lunatus	2.53
G_oc-temp_med-Parahip	3.54	S_oc_sup_and_transversal	2.67
G_orbital	3.19	S_occipital_ant	2.70
G_pariet_inf-Angular	3.18	S_oc-temp_lat	2.83
G_pariet_inf-Supramar	3.14	S_oc-temp_med_and_Lingual	2.83
G_parietal_sup	2.68	S_orbital_lateral	2.75
G_postcentral	2.54	S_orbital_med-olfact	2.76
G_precentral	3.07	S_orbital-H_Shaped	3.11
G_precuneus	2.96	S_parieto_occipital	2.60
G_rectus	2.87	S_pericallosal	1.90
G_subcallosal	2.79	S_postcentral	2.70
G_temp_sup-G_T_transv	2.82	S_precentral-inf-part	2.79
G_temp_sup-Lateral	3.47	S_precentral-sup-part	2.71
G_temp_sup-Plan_polar	3.43	S_suborbital	2.81
G_temp_sup-Plan_tempo	2.99	S_subparietal	3.21
G_temporal_inf	3.38	S_temporal_inf	3.08
G_temporal_middle	3.43	S_temporal_sup	2.91
		S_temporal_transverse	3.01
Avg.	3.04±0.94		2.75±0.58

Table S4 The averaged cortical thickness (mm) of gyri and sulci on subject #4.

Gyri	Thickness	Sulci	Thickness
G_cingul-Post-dorsal	3.10	S_calcarine	2.13
G_cingul-Post-ventral	2.22	S_central	1.86
G_cuneus	2.04	S_cingul-Marginalis	2.30
G_front_inf-Opercular	2.82	S_circular_insula_ant	2.78
G_front_inf-Orbital	2.82	S_circular_insula_inf	2.53
G_front_inf-Triangul	2.66	S_circular_insula_sup	2.66

G_front_middle	2.73	S_collat_transv_ant	2.63
G_front_sup	2.84	S_collat_transv_post	2.19
G_Ins_lg_and_S_cent_ins	3.21	S_front_inf	2.49
G_insular_short	3.75	S_front_middle	2.37
G_occipital_middle	2.63	S_front_sup	2.40
G_occipital_sup	2.31	S_interm_prim-Jensen	2.19
G_oc-temp_lat-fusifor	3.09	S_intrapariet_and_P_trans	2.24
G_oc-temp_med-Lingual	2.09	S_oc_middle_and_Lunatus	2.08
G_oc-temp_med-Parahip	3.12	S_oc_sup_and_transversal	2.23
G_orbital	2.71	S_occipital_ant	2.39
G_pariet_inf-Angular	2.67	S_oc-temp_lat	2.63
G_pariet_inf-Supramar	2.73	S_oc-temp_med_and_Lingual	2.40
G_parietal_sup	2.33	S_orbital_lateral	2.20
G_postcentral	2.20	S_orbital_med-olfact	2.42
G_precentral	2.72	S_orbital-H_Shaped	2.61
G_precuneus	2.61	S_parieto_occipital	2.20
G_rectus	2.64	S_pericallosal	1.98
G_subcallosal	2.82	S_postcentral	2.07
G_temp_sup-G_T_transv	2.73	S_precentral-inf-part	2.51
G_temp_sup-Lateral	3.13	S_precentral-sup-part	2.30
G_temp_sup-Plan_polar	3.29	S_suborbital	2.48
G_temp_sup-Plan_tempo	2.75	S_subparietal	2.46
G_temporal_inf	3.14	S_temporal_inf	2.69
G_temporal_middle	3.03	S_temporal_sup	2.57
		S_temporal_transverse	2.90
Avg.	2.76±0.99		2.38±0.52

Table S5 The averaged cortical thickness (mm) of gyri and sulci on subject #5.

**Gyri**	**Thickness**	**Sulci**	**Thickness**
G_cingul-Post-dorsal	2.96	S_calcarine	2.12
G_cingul-Post-ventral	2.49	S_central	2.03
G_cuneus	2.15	S_cingul-Marginalis	2.43
G_front_inf-Opercular	3.20	S_circular_insula_ant	2.97
G_front_inf-Orbital	2.89	S_circular_insula_inf	2.93
G_front_inf-Triangul	3.00	S_circular_insula_sup	3.00
G_front_middle	2.88	S_collat_transv_ant	2.74
G_front_sup	3.07	S_collat_transv_post	2.21
G_Ins_lg_and_S_cent_ins	3.65	S_front_inf	2.50
G_insular_short	3.97	S_front_middle	2.53
G_occipital_middle	2.75	S_front_sup	2.72
G_occipital_sup	2.37	S_interm_prim-Jensen	2.50
G_oc-temp_lat-fusifor	3.00	S_intrapariet_and_P_trans	2.31
G_oc-temp_med-Lingual	2.21	S_oc_middle_and_Lunatus	2.15
G_oc-temp_med-Parahip	3.33	S_oc_sup_and_transversal	2.37

G_orbital	2.95	S_occipital_ant	2.60
G_pariet_inf-Angular	2.82	S_oc-temp_lat	2.77
G_pariet_inf-Supramar	2.79	S_oc-temp_med_and_Lingual	2.34
G_parietal_sup	2.55	S_orbital_lateral	2.40
G_postcentral	2.35	S_orbital_med-olfact	2.32
G_precentral	2.95	S_orbital-H_Shaped	2.71
G_precuneus	2.94	S_parieto_occipital	2.38
G_rectus	2.61	S_pericallosal	2.14
G_subcallosal	2.25	S_postcentral	2.31
G_temp_sup-G_T_transv	2.55	S_precentral-inf-part	2.75
G_temp_sup-Lateral	3.32	S_precentral-sup-part	2.63
G_temp_sup-Plan_polar	3.74	S_suborbital	2.81
G_temp_sup-Plan_tempo	2.57	S_subparietal	2.71
G_temporal_inf	3.22	S_temporal_inf	2.84
G_temporal_middle	3.24	S_temporal_sup	2.78
		S_temporal_transverse	2.34
Avg.	2.88±1.09		2.53±0.49

Table S6 The averaged cortical thickness (mm) of gyri and sulci on subject #6.

**Gyri**	**Thickness**	**Sulci**	**Thickness**
G_cingul-Post-dorsal	3.08	S_calcarine	2.25
G_cingul-Post-ventral	2.18	S_central	2.05
G_cuneus	2.09	S_cingul-Marginalis	2.38
G_front_inf-Opercular	3.13	S_circular_insula_ant	3.50
G_front_inf-Orbital	3.35	S_circular_insula_inf	2.63
G_front_inf-Triangul	2.95	S_circular_insula_sup	2.94
G_front_middle	2.85	S_collat_transv_ant	2.84
G_front_sup	3.01	S_collat_transv_post	2.37
G_Ins_lg_and_S_cent_ins	3.32	S_front_inf	2.67
G_insular_short	3.79	S_front_middle	2.60
G_occipital_middle	2.78	S_front_sup	2.70
G_occipital_sup	2.29	S_interm_prim-Jensen	2.46
G_oc-temp_lat-fusifor	3.23	S_intrapariet_and_P_trans	2.33
G_oc-temp_med-Lingual	2.39	S_oc_middle_and_Lunatus	2.22
G_oc-temp_med-Parahip	3.40	S_oc_sup_and_transversal	2.31
G_orbital	2.93	S_occipital_ant	2.72
G_pariet_inf-Angular	2.81	S_oc-temp_lat	2.82
G_pariet_inf-Supramar	2.87	S_oc-temp_med_and_Lingual	2.79
G_parietal_sup	2.39	S_orbital_lateral	2.59
G_postcentral	2.37	S_orbital_med-olfact	2.66
G_precentral	2.78	S_orbital-H_Shaped	2.78
G_precuneus	2.72	S_parieto_occipital	2.48
G_rectus	2.71	S_pericallosal	1.55
G_subcallosal	2.75	S_postcentral	2.27

G_temp_sup-G_T_transv	2.83	S_precentral-inf-part	2.63
G_temp_sup-Lateral	3.23	S_precentral-sup-part	2.52
G_temp_sup-Plan_polar	3.40	S_suborbital	2.82
G_temp_sup-Plan_tempo	2.75	S_subparietal	2.50
G_temporal_inf	3.12	S_temporal_inf	2.85
G_temporal_middle	3.24	S_temporal_sup	2.76
		S_temporal_transverse	2.68
Avg.	2.88±0.91		2.57±1.02

Table S7 The averaged cortical thickness (mm) of gyri and sulci on subject #7.

**Gyri**	**Thickness**	**Sulci**	**Thickness**
G_cingul-Post-dorsal	3.22	S_calcarine	2.29
G_cingul-Post-ventral	2.25	S_central	2.21
G_cuneus	2.19	S_cingul-Marginalis	2.57
G_front_inf-Opercular	3.17	S_circular_insula_ant	3.10
G_front_inf-Orbital	3.29	S_circular_insula_inf	2.95
G_front_inf-Triangul	2.98	S_circular_insula_sup	3.01
G_front_middle	2.98	S_collat_transv_ant	2.90
G_front_sup	3.11	S_collat_transv_post	2.34
G_Ins_lg_and_S_cent_ins	3.52	S_front_inf	2.63
G_insular_short	4.04	S_front_middle	2.70
G_occipital_middle	3.02	S_front_sup	2.79
G_occipital_sup	2.65	S_interm_prim-Jensen	2.90
G_oc-temp_lat-fusifor	3.13	S_intrapariet_and_P_trans	2.49
G_oc-temp_med-Lingual	2.29	S_oc_middle_and_Lunatus	2.49
G_oc-temp_med-Parahip	3.20	S_oc_sup_and_transversal	2.52
G_orbital	3.05	S_occipital_ant	2.70
G_pariet_inf-Angular	3.13	S_oc-temp_lat	2.91
G_pariet_inf-Supramar	3.16	S_oc-temp_med_and_Lingual	2.70
G_parietal_sup	2.77	S_orbital_lateral	2.32
G_postcentral	2.55	S_orbital_med-olfact	2.39
G_precentral	3.08	S_orbital-H_Shaped	2.99
G_precuneus	2.92	S_parieto_occipital	2.52
G_rectus	2.73	S_pericallosal	1.84
G_subcallosal	3.14	S_postcentral	2.55
G_temp_sup-G_T_transv	2.97	S_precentral-inf-part	2.75
G_temp_sup-Lateral	3.39	S_precentral-sup-part	2.77
G_temp_sup-Plan_polar	3.54	S_suborbital	2.89
G_temp_sup-Plan_tempo	2.80	S_subparietal	2.72
G_temporal_inf	3.29	S_temporal_inf	2.87
G_temporal_middle	3.38	S_temporal_sup	2.89
		S_temporal_transverse	2.95
Avg.	3.02±1.02		2.67±0.82

Table S8 The averaged cortical thickness (mm) of gyri and sulci on subject #8.

Gyri	Thickness	Sulci	Thickness
G_cingul-Post-dorsal	3.16	S_calcarine	2.15
G_cingul-Post-ventral	2.62	S_central	2.16
G_cuneus	2.02	S_cingul-Marginalis	2.51
G_front_inf-Opercular	3.03	S_circular_insula_ant	3.23
G_front_inf-Orbital	3.10	S_circular_insula_inf	2.78
G_front_inf-Triangul	2.89	S_circular_insula_sup	2.84
G_front_middle	2.85	S_collat_transv_ant	2.83
G_front_sup	3.09	S_collat_transv_post	2.33
G_Ins_lg_and_S_cent_ins	3.19	S_front_inf	2.47
G_insular_short	3.48	S_front_middle	2.45
G_occipital_middle	2.86	S_front_sup	2.82
G_occipital_sup	2.47	S_interm_prim-Jensen	2.68
G_oc-temp_lat-fusifor	3.04	S_intrapariet_and_P_trans	2.54
G_oc-temp_med-Lingual	2.12	S_oc_middle_and_Lunatus	2.43
G_oc-temp_med-Parahip	2.91	S_oc_sup_and_transversal	2.30
G_orbital	2.91	S_occipital_ant	2.72
G_pariet_inf-Angular	3.08	S_oc-temp_lat	2.74
G_pariet_inf-Supramar	2.99	S_oc-temp_med_and_Lingual	2.60
G_parietal_sup	2.59	S_orbital_lateral	2.16
G_postcentral	2.49	S_orbital_med-olfact	2.33
G_precentral	3.06	S_orbital-H_Shaped	2.70
G_precuneus	2.82	S_parieto_occipital	2.36
G_rectus	2.74	S_pericallosal	1.75
G_subcallosal	3.07	S_postcentral	2.48
G_temp_sup-G_T_transv	2.90	S_precentral-inf-part	2.67
G_temp_sup-Lateral	3.25	S_precentral-sup-part	2.68
G_temp_sup-Plan_polar	3.46	S_suborbital	2.92
G_temp_sup-Plan_tempo	2.73	S_subparietal	2.62
G_temporal_inf	3.07	S_temporal_inf	2.87
G_temporal_middle	3.36	S_temporal_sup	2.80
		S_temporal_transverse	2.58
Avg.	2.90±0.88		2.56±0.82

Table S9 The averaged cortical thickness (mm) of gyri and sulci on subject #9.

Gyri	Thickness	Sulci	Thickness
G_cingul-Post-dorsal	3.18	S_calcarine	2.16
G_cingul-Post-ventral	2.36	S_central	2.07
G_cuneus	2.20	S_cingul-Marginalis	2.40
G_front_inf-Opercular	2.98	S_circular_insula_ant	3.06
G_front_inf-Orbital	3.14	S_circular_insula_inf	2.60

G_front_inf-Triangul	2.82	S_circular_insula_sup	2.81
G_front_middle	2.93	S_collat_transv_ant	2.90
G_front_sup	3.09	S_collat_transv_post	2.26
G_Ins_lg_and_S_cent_ins	3.30	S_front_inf	2.64
G_insular_short	3.51	S_front_middle	2.56
G_occipital_middle	2.78	S_front_sup	2.72
G_occipital_sup	2.39	S_interm_prim-Jensen	2.62
G_oc-temp_lat-fusifor	3.24	S_intrapariet_and_P_trans	2.40
G_oc-temp_med-Lingual	2.41	S_oc_middle_and_Lunatus	2.40
G_oc-temp_med-Parahip	3.08	S_oc_sup_and_transversal	2.36
G_orbital	2.92	S_occipital_ant	2.75
G_pariet_inf-Angular	2.95	S_oc-temp_lat	2.74
G_pariet_inf-Supramar	2.93	S_oc-temp_med_and_Lingual	2.57
G_parietal_sup	2.57	S_orbital_lateral	2.55
G_postcentral	2.35	S_orbital_med-olfact	2.38
G_precentral	3.00	S_orbital-H_Shaped	2.89
G_precuneus	2.82	S_parieto_occipital	2.38
G_rectus	2.86	S_pericallosal	1.65
G_subcallosal	2.61	S_postcentral	2.31
G_temp_sup-G_T_transv	2.95	S_precentral-inf-part	2.71
G_temp_sup-Lateral	3.11	S_precentral-sup-part	2.55
G_temp_sup-Plan_polar	3.12	S_suborbital	2.60
G_temp_sup-Plan_tempo	2.89	S_subparietal	2.53
G_temporal_inf	3.02	S_temporal_inf	2.65
G_temporal_middle	3.15	S_temporal_sup	2.63
		S_temporal_transverse	2.88
Avg.	2.88±0.68		2.54±0.89

Table S10 The averaged cortical thickness (mm) of gyri and sulci on subject #10.

**Gyri**	**Thickness**	**Sulci**	**Thickness**
G_cingul-Post-dorsal	3.06	S_calcarine	2.13
G_cingul-Post-ventral	2.21	S_central	2.13
G_cuneus	2.45	S_cingul-Marginalis	2.53
G_front_inf-Opercular	2.96	S_circular_insula_ant	3.27
G_front_inf-Orbital	3.07	S_circular_insula_inf	2.67
G_front_inf-Triangul	2.98	S_circular_insula_sup	2.91
G_front_middle	2.85	S_collat_transv_ant	3.08
G_front_sup	3.17	S_collat_transv_post	2.30
G_Ins_lg_and_S_cent_ins	2.96	S_front_inf	2.67
G_insular_short	3.52	S_front_middle	2.54
G_occipital_middle	2.95	S_front_sup	2.74
G_occipital_sup	2.58	S_interm_prim-Jensen	2.71
G_oc-temp_lat-fusifor	3.03	S_intrapariet_and_P_trans	2.56
G_oc-temp_med-Lingual	2.32	S_oc_middle_and_Lunatus	2.42

G_oc-temp_med-Parahip	3.11	S_oc_sup_and_transversal	2.47
G_orbital	2.92	S_occipital_ant	2.53
G_pariet_inf-Angular	3.10	S_oc-temp_lat	2.78
G_pariet_inf-Supramar	3.10	S_oc-temp_med_and_Lingual	2.69
G_parietal_sup	2.63	S_orbital_lateral	2.69
G_postcentral	2.55	S_orbital_med-olfact	2.47
G_precentral	3.10	S_orbital-H_Shaped	2.69
G_precuneus	2.89	S_parieto_occipital	2.51
G_rectus	2.64	S_pericallosal	1.69
G_subcallosal	2.87	S_postcentral	2.53
G_temp_sup-G_T_transv	2.83	S_precentral-inf-part	2.74
G_temp_sup-Lateral	3.15	S_precentral-sup-part	2.67
G_temp_sup-Plan_polar	3.03	S_suborbital	2.83
G_temp_sup-Plan_tempo	3.02	S_subparietal	2.64
G_temporal_inf	3.05	S_temporal_inf	2.70
G_temporal_middle	3.21	S_temporal_sup	2.73
		S_temporal_transverse	2.80
Avg.	2.90±0.69		2.61±0.61

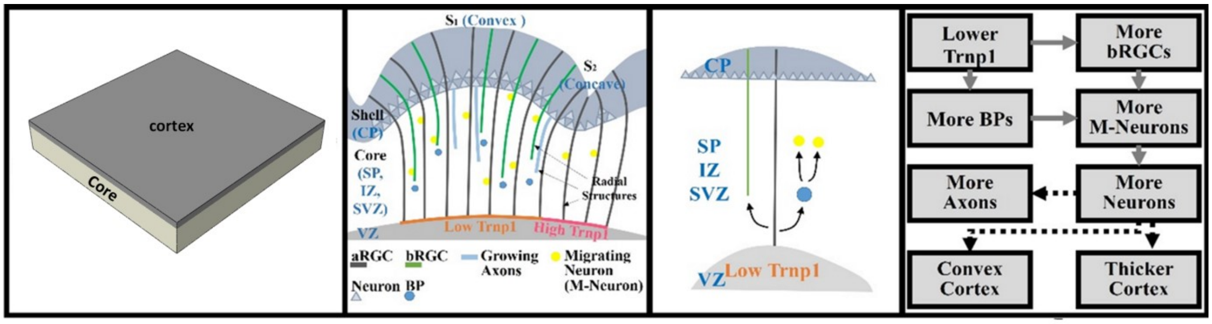

(a) (b) (c) (d)

Figure S1. (a) An idealized 3D model for a small piece of the brain; (b) and (c) biological viewpoints of neurogenesis; (d) flow chart of how Trnp1 regulates the cortical folding patterns. The dashed line arrows suggest the postulated gyrification process at the macro-scale. Abbreviations: RGC, radial glial cell; aRGC, apical RGC; bRGC, basal RGC; BP, basal progenitor; CP, cortical plate; VZ, ventricular zone; SVZ, subventricular zone; SP, subplate; IZ, intermediate zone.

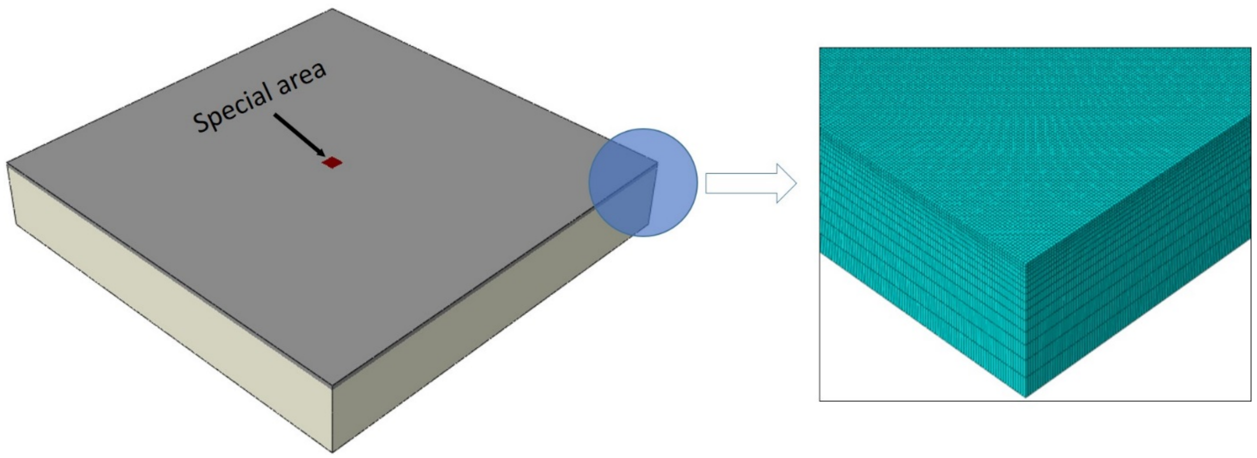

Figure S2. A 3D planar double-layered brain model with a fine mesh in the top cortex layer.
